# Scientific Items

**Published:** 1883-01-20

**Authors:** 


### SCIENTIFIC ITEMS.

**Misers.**—It is remarkable that misers generally are long lived. Many years ago Samuel Bailey, a farmer in the Isle of Wight, subjected himself and family to incredible privations. In order to save feed for horses they even did the ploughing and harrowing themselves, and would eat the flesh of animals which had died a natural death. Yet he lived to be 92.—*Microcosm.*

**Vanadium**, discovered in 1801 by Del Rio, existed up to 1867 as one of the rarest of chemical curiosities. At that time a Rouen calico printer made some experiments with it, and found it so superior to the sulphide of copper that it grew into demand. But vanadium was so difficult to obtain that even in 1867 its compounds, containing but 50 per cent. of the metal, were equal to the price of gold. Within the past year, however, large quantities were found in France, in alkaline earths, and the supply is supposed to be adequate to all demands. This is a striking instance of the unexpected practical value often found in products originally supposed to be of interest only to theoretical chemists.—*Microcosm.*

**Submarine Work.**—Thanks to the enterprise of an English firm, the submarine diver of the future is likely to have all the advantages which Jules Verne gave to the divers in his wonderful work, "Twenty Thousand Leagues Under the Sea." Our readers will remember that there the divers are represented by the author as putting on their armor in the usual fashion, and then attaching oxygen reservoirs at the back; going into the water free and independent of air-pumps and heavy-dragging air-pipes. The new system calls for the manufacture of oxygen, and its compression into tanks, which are strapped upon the armor. The carbonic acid of the breath is removed by means of caustic potash, and a fresh supply of oxygen takes the place of that used up by breathing. The diver is of course entirely independent of the surface, and can walk about as much at his ease as it is possible beneath the surface of the water, weighted by the usual amount of lead necessary to keep him submerged.—*Boston Jour. Chemistry.*

**Australian Big Trees.**—A foreign exchange says: "The trackless forests in the west of Tasmania contain huge timber, and bushmen report that they have met with specimens of Eucalyptus measuring 200 feet from the ground to the first branch, and fully 350 feet in all. Until 1873 there was standing on the eastern slope of Mount Wellington, within four miles of Hobart Town, a Eucalyptus which measured 86 feet in girth and more than 300 feet in height, and its ruined boll still forms a grim chamber, in which many a merry party have joined a picnic. The famous tree of the Huon Forest measures 70 feet in girth 6 feet from the ground, and is stated to be 240 feet high; but in the deep gorges of this grand forest the writer has seen higher trees than this, though not of quite equal circumference. Victoria, however, now claims the glory of holding the biggist of all the living 'big trees' in the world, so far as height is concerned. In the Dandenong district at Fernshaw has recently been discovered a specimen of Eucalyptus amygdalina, or almond-leaf gum, which, accurately measured, reached the enormous height of 380 feet before throwing out a single branch, and 430 feet to the top, and having a girth of 60 feet at some distance above the ground." Some idea of what an elevation of 430 feet represents may be gained from the fact that this gum-tree, if growing by the side of the Bunker Hill Monument, would stand almost twice as high as that lofty obelisk, which is 220 feet in height.—*Boston Journal Chemistry.*

**Hurricanes.**—Captain A. W. Jeffrey, commander of the British steamship Ptolemy, who is engaged in making regular meteorological observations for his own government and the Signal Service of the United States, has of late been looking into the causes which produce West India hurricanes. He is of the opinion that the prime disturbing power is located far to the eastward of where the storm bursts on the surface of the globe and sweeps to the westward, being so dangerous to navigation and harmful to the shipping interest. Captain Jeffrey has watched closely the action and movements of the upper clouds in the equatorial latitudes for years, and finds that the upper currents are disturbed far to the eastward of true hurricane development. He traces the cause to the intense tropical heat poured down on the African deserts, whereby vacuums in the atmosphere are formed by the hot air rising; the air expands and flows off literally, the equilibrium of the mass is disturbed and drifts to the westward with the currents growing in size and obtaining more energy until the lower currents intermingle and a hurricane is formed. There has been forwarded by Captain Jeffrey to General Hazen, Chief Signal Officer of the army, a communication in reference to this matter, giviug the facts and figures in detail.—*Mechanical News.*

**The Moon and the Weather.**—At the meeting of the British Association, Sir W. Thompson delivered an address to a large audience upon the tides. While explaining the theory of the moon's influence on the tides, he incidentally touched on the supposed influence of the moon's changes upon weather, and pointed out that the comparison of most careful and complete indications of the barometer, thermometer and anemometer, and the times of the new and full moon and half moon, had failed to establish any relation whatever between them, and had proved, on the contrary, that if there was any dependence of the weather on the phases of the moon it was only to a degree quite imperceptible to ordinary observation. We might take it confidently not only that it was not proved that there was a dependence of the weather on the changes of the moon, but that it was proved that there was no general dependence of weather on the changes of the moon.—*Boston Journal Chemistry.*

